# Factors related with nursing students’ health literacy: a cross sectional study

**DOI:** 10.3389/fpubh.2023.1053016

**Published:** 2023-05-18

**Authors:** Enrique Ramón-Arbués, José Manuel Granada-López, Isabel Antón-Solanas, Ana Cobos-Rincón, Antonio Rodríguez-Calvo, Vicente Gea-Caballero, Clara Isabel Tejada-Garrido, Raúl Juárez-Vela, Emmanuel Echániz-Serrano

**Affiliations:** ^1^Faculty of Health Sciences, San Jorge University, Villanueva de Gállego, Spain; ^2^SAPIENF Investigation Group, Zaragoza, Spain; ^3^Department of Nursing and Physiatry, Faculty of Health Sciences, University of Zaragoza, Zaragoza, Spain; ^4^GIISA021 Seguridad y Cuidados Investigation Group, Zaragoza, Spain; ^5^Department of Nursing, Faculty of Health Sciences, University of La Rioja, Logroño, Spain; ^6^Biomedical Research Center of La Rioja, CIBIR, Logroño, Spain; ^7^Department of Anesthesia, Complex University of Salamanca, Salamanca, Spain; ^8^Faculty of Medicine, University of Salamanca, Salamanca, Spain; ^9^Faculty of Health Sciences, Valencian International University, Valencia, Spain; ^10^Community Health and Care Research Group, SALCOM, Valencia, Spain

**Keywords:** nursing education, health literacy, HLS-EU-Q16, health promotion, health behavior

## Abstract

**Background:**

Nursing professionals have a crucial role in promoting health literacy in health services, so it is necessary to ensure health literacy skills in future health professionals.

**Objective:**

The objective of the study was to examine the health literacy of nursing students and its associated factors.

**Methods:**

A cross-sectional descriptive study was carried out on 460 nursing students. For data collection, a semi-structured questionnaire was obtained on sociodemographic characteristics, perception and health care, use of the health system and lifestyles. In addition, health literacy was assessed using the European Health Literacy Questionnaire.

**Results:**

6.1% of the participants had an inadequate level of health literacy and 36.5% problematic. The probability of having sufficient health literacy is directly associated with age; and inversely with smoking, prolonged screen time and living alone (*p* < 0.05).

**Conclusion:**

A large percentage of nursing students need to improve their health literacy skills. It is necessary to integrate a greater number of contents in health literacy in the curriculum of nursing students.

## Introduction

The concept of Health Literacy (HL) was introduced in the study of Public Health 4 decades ago. Since then, it has become an increasingly relevant topic and a critical determinant of health (1). It is a complex concept that has given rise to numerous definitions over time (2, 3). In most of them, it is considered as a set of individual capabilities that allow the person to acquire and apply information in self-care and in decision-making about health care (4). In this way, HL includes the understanding and evaluation of the sufficiency of information related to health, the correct use of medications, the use of health services, informed consent forms; as well as decision-making about self-care and disease management (5). In contrast, poor HL can lead to increased drug misuse (6), unhealthy behaviors (7), deficiencies in disease management (8) and access to care services that ultimately can lead to increased morbidity and mortality (9, 10), a deterioration in personal quality of life (11) and an increase in social costs (12).

Worldwide, the review of the level of HL in the general population suggests wide margins for improvement (13, 14). At present there are mainly three, the ways that people use to find out about health; the media and the Internet, peers and health professionals (15). Under this prism, the training in HL of health personnel during their training is of vital importance, especially in the case of future nurses who will ultimately be the main figures in health education. Thus, adequate HL can benefit the working life of future nurses by improving their communication with patients, reducing gaps in patient knowledge about their disease, improving adherence to treatment, and consequently reducing morbidity, mortality and medical treatment failure (4). Along these lines, Toronto and Weatherfort (16) in their review of the presence and characteristics of HL included in training programs for health professionals pointed out the need to assess students’ HL through validated instruments.

Previous literature on the level of HL in nursing students seems to indicate a wide margin for improvement (7, 17, 18), with figures around 30% of students with problematic or limited HL (19, 20). However, the body of knowledge is still scarce in relation to the subject, especially in the Spanish context, requiring new research. In this context, the objective of this study was to evaluate the level of HL in a cohort of nursing students and to explore its association with their perception and health care, use of the health system, sociodemographic characteristics, and lifestyles.

## Method

### Design

A cross-sectional descriptive study was carried out. The reference population was Nursing students from the Faculty of Health Sciences of the San Jorge University in Villanueva de Gállego, Aragón (Spain).

### Sample

**Figure 1:**
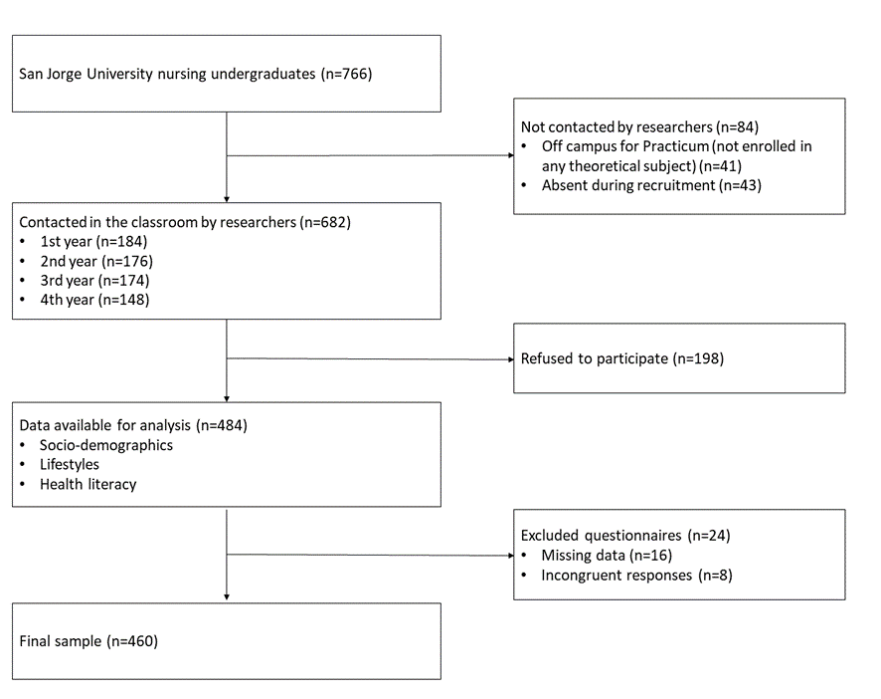
Study flow-chart.

The recruitment of participants and data collection was carried out in the classrooms during the months of September to December 2021. A researcher informed the students in their respective classrooms about the objectives of the study and the methods of data collection. At this time, students were given a study information sheet and informed consent document. Students were assured that privacy and confidentiality would be maintained, and that they had the right to refuse to participate in the study or withdraw their consent to participate at any time. Of an enrolled population of 682, 484 gave their consent to participate in this study and completed the requested questionnaires. Of the 484 questionnaires received, 24 were considered invalid (generalized non-completion or manifestly unrealistic data) and therefore were excluded from the analysis (Figure 1).

### Data collection

The data collection questionnaire was made up of three sections: 1. sociodemographic data and lifestyles; 2. health, perceived health and use of care resources and 3. HL. Thus, the participants reported information on their age (≤20 years; 21–29 years; ≥30 years), gender (male; female), living arrangement (alone; with mates; with family), employment status (employed; unemployed), tobacco use (yes; no), screen-time (≤4 h/day; 5–7 h/day; ≥8 h/day), alcohol consumption (never or occasionally; at least once a week), perceived general health (bad health; good health), chronic disorders (yes; no), self-medication last week (yes; no) and healthcare utilization last year (0–1 visits; 2–7 visits; ≥8 visits). In addition, validated questionnaires were used to assess physical activity, diet quality, and HL.

Physical activity was assessed using the International Physical Activity Questionnaire-Short form (IPAQ-SF). IPAQ-SF provides information about the intensity, frequency and duration of physical activity performed in the last 7 days. Based on the information collected, the population can be classified by categories of physical activity developed. Namely: high, moderate and low (21). This questionnaire has been validated for the Spanish university population, showing a satisfactory correlation (0.69) with the accelerometer (22).

The participants’ diet was evaluated using the Spanish Healthy Eating Index (HEI) (23). It is an adaptation of the Healthy Eating Index by Kennedy et al. (24) to the Spanish context according to the recommendations of the Spanish Society of Community Nutrition. The SHEI consists of 10 items (score range 0–10). The final score in this questionnaire ranges between 0 and 100 points and categorizes the participants based on the following criteria: score > 80 (healthy diet), between 50 and 80 points (need changes) and < 50 points (inadequate diet). The HEI has been validated through plasmatic biomarkers showing satisfactory correlation levels ranging from a minimum of *r* = 0.23 (for grains) to a maximum of *r* = 0.71 for the food variety (25).

For the evaluation of HL, the 16-item European Health-literacy Questionnaire (HLS-EU-Q16) (13) was used. This is an instrument, developed by the HLS-EU Consortium and validated in the Spanish population (26), which assesses three domains (“health care,” “disease prevention,” and “health promotion”). In this questionnaire, each respondent classifies the degree of difficulty perceived for each task or situation as “very easy,” “easy,” “difficult,” or “very difficult.” In order to discriminate the level of literacy of the participants, the researcher dichotomizes the responses into “very difficult or difficult” = 0, and “easy or very easy” = 1. From these scores, the subjects can be categorized as inadequate HL (1–8 points), problematic HL (9–12 points) and sufficient HL (13–16 points) (27).

### Analysis of data

The results of the descriptive analysis are presented through the mean and its standard deviation for continuous variables and the number and percentage for categorical variables. The bivariate analysis between the different variables and belonging to a certain level of HL was performed using the χ^2^-test. Finally, a binary logistic regression model (enter method) was carried out to predict the probability of sufficient level of HL. Data coding, processing and analysis were completed using the statistical software Statistical Package for the Social Science (SPSS version 21 for Windows, IBM Corp., Chicago, IL, United States) accepting a level of significance of *p* < 0.05.

### Ethical considerations

The research protocol was reviewed and approved by the Clinical Research Ethics Committee of Aragón prior to starting the study. In addition, the undersigned authors confirm that each and every one of the national and international standards of ethical research with human beings was respected and complied with at all times.

## Results

The questionnaire was completed by 460 subjects, predominantly female (86.1%), age ≤ 20 years (64.3%), residents of the family home (56.5%) and those who did not have a paid job (84.3%). Regarding their health and their care, a majority reported being in good health (93.0%), not presenting any chronic disease (77.4%) and not regularly self-medicating (76.5%). The average use of health services was 4.7 ± 4.9 health care received with a median of 3(IQR 2–6). The prevalence of lifestyles negative for health was 38.3% of smokers and 35.7% of regular alcohol consumers. In addition, 45.2% of the subjects reported low physical activity and up to 92.2% a diet in need of changes (Table 1).

**Table 1:** Participant characteristics (*n* = 460).

		Inadequate HL	Problematic HL	Sufficient HL	*p*-value^*^
	n (%)	n (%)	n (%)	n (%)	
Total	460 (100%)	28 (6.1%)	168 (36.5%)	256 (57.4%)	
Age groups					
≤20 years	296 (64.3%)	24 (8.1%)	116 (39.2%)	156 (52.7%)	0.000
21–29 years	84 (18.3%)	4 (4.8%)	40 (47.6%)	40 (47.6%)	
≥30 years	80 (17.4%)	0 (0.0%)	12 (15.0%)	68 (85.0%)	
Gender					
Male	64 (13.9%)	4 (6.3%)	12 (18.8%)	48 (75.0%)	0.005
Female	396 (86.1%)	24 (6.1%)	156 (39.4%)	216 (54.5%)	
Living arrangement					
Living alone	36 (7.8%)	4 (11.1%)	16 (44.4%)	16 (44.4%)	0.380
Living with mates	164 (35.7%)	8 (4.9%)	56 (34.1%)	100 (61.0%)	
Living with family	260 (56.5%)	16 (6.2%)	96 (36.9%)	148 (56.9%)	
Employment status					
Employed	72 (15.7%)	0 (0.0%)	24 (33.3%)	48 (66.7%)	0.035
Unemployed	388 (84.3%)	28 (7.2%)	144 (37.1%)	216 (55.7%)	
Perceived general health					
Bad health	32 (7.0%)	4 (12.5%)	8 (25.0%)	20 (62.5%)	0.155
Good health	428 (93.0%)	24 (5.6%)	160 (37.4%)	244 (57.0%)	
Chronic disorder					
Yes	104 (22.6%)	8 (7.7%)	36 (34.6%)	60 (57.7%)	0.703
No	356 (77.4%)	20 (5.6%)	132 (37.1%)	204 (57.3%)	
Healthcare utilization last year					
0–1 visits	92 (20.0%)	8 (8.7%)	28 (30.4%)	56 (60.9%)	0.339
2–7 visits	292 (63.5%)	16 (5.5%)	116 (39.7%)	160 (54.8%)	
≥8 visits	76 (16.5%)	4 (5.3%)	24 (31.6%)	48 (63.2%)	
Self-medication last week					
Yes	108 (23.5%)	8 (7.4%)	40 (37.0%)	60 (55.6%)	0.780
No	352 (76.5%)	20 (5.7%)	128 (36.4%)	204 (58.0%)	
Tobacco use					
Yes	176 (38.3%)	12 (6.8%)	76 (43.2%)	88 (50.0%)	0.040
No	284 (61.7%)	16 (5.6%)	92 (32.4%)	176 (62.0%)	
Alcohol consumption					
Never or occasionally	296 (64.3%)	12 (4.1%)	108 (36.5%)	176 (59.5%)	0.044
At least once a week	164 (35.7%)	16 (9.8%)	60 (36.6%)	88 (53.7%)	
Screen-time					
≤4 h per day	96 (20.9%)	4 (4.2%)	20 (20.8%)	72 (75.0%)	0.001
5–7 h per day	268 (58.2%)	20 (7.5%)	112 (41.8%)	136 (50.7%)	
≥8 h per day	96 (20.9%)	4 (4.2%)	36 (37.5%)	56 (58.3%)	
Diet quality					
Inadequate	52 (11.3%)	4 (7.6%)	24 (46.2%)	24 (46.2%)	0.048
Need changes	372 (80.9%)	24 (6.5%)	136 (36.6%)	212 (57.0%)	
Healthy	36 (7.8%)	0 (0.0%)	8 (22.2%)	28 (77.8%)	
Physical activity					
Low	208 (45.2%)	8 (3.8%)	68 (32.7%)	132 (63.5%)	0.067
Medium	132 (28.7%)	12 (9.1%)	48 (36.4%)	72 (54.5%)	
High	120 (26.1%)	8 (6.7%)	52 (43.3%)	60 (50.0%)	

^*^X^2^-test.

In relation to the results of the HLS-EU-Q16, 57.4, 36.5, and 6.1%, respectively, reported an adequate, problematic and inadequate HL (Table 1). In the bivariate analysis, the factors associated with sufficient HL were age ≥ 30 years, male gender, and adherence to healthy lifestyles (non-smoking, limited alcohol consumption, low screen-time, and adherence to a healthy diet) (Table 1). The best satisfied dimension of HL in our sample was “Health Promotion” and the worst was “Disease prevention.” Table 2 summarizes the responses of the participants to each item.

**Table 2:** Results of HLS-EU-Q16 scores in the total sample (*n* = 460).

Domain	Item	Very difficult	Difficult	Easy	Very easy	Mean ± SD (item)^*^	Mean ± SD (domain)
Health care	(1) —find information on treatments or illnesses that concern you?	0 (0%)	44 (9.6%)	52 (11.3%)	364 (79.1%)	3.01 ± 0.45	3.05 ± 0.68
	(2) —find out where to get professional help when you are ill?	0 (0%)	20 (4.3%)	232 (50.5%)	208 (45.2%)	3.40 ± 0.57	
	(3) —understand what the doctor says to you?	0 (0%)	72 (15.6%)	304 (66.1%)	84 (18.3%)	3.02 ± 0.58	
	(4) —understand your doctor’s or pharmacist’s instructions on how to take a prescribed medicine?	0 (0%)	24 (5.2%)	240 (52.2%)	196 (42.6%)	3.37 ± 0.58	
	(5) —judge when you need to get a second opinion from your doctor?	28 (6.1%)	220 (47.9%)	176 (38.2%)	36 (7.8%)	2.47 ± 0.72	
	(6) —use information the doctor gives you to make decisions about your illness?	12 (2.6%)	164 (35.7%)	252 (54.8%)	32 (6.9%)	2.66 ± 0.64	
	(7) —follow instructions from your doctor or pharmacist?	4 (0.9%)	0 (0%)	244 (53.0%)	212 (46.1%)	3.44 ± 0.54	
Disease prevention	(8) —find information on how to manage mental health problems like stress or depression?	28 (6.1%)	156 (33.9%)	220 (47.8%)	56 (12.2%)	2.66 ± 0.76	3.00 ± 1.41
	(9) —understand health warnings about behavior such as smoking, low physical activity and drinking too much?	0 (0%)	44 (9.6%)	52 (11.3%)	364 (79.1%)	3.43 ± 0.60	
	(10) —understand why you need health screenings?	0 (0%)	12 (2.6%)	192 (41.7%)	256 (55.7%)	3.53 ± 0.54	
	(11) —judge if the information on health risks in the media is reliable?	32 (6.9%)	160 (34.8%)	232 (50.5%)	36 (7.8%)	2.59 ± 0.73	
	(12) —decide how you can protect yourself from illness based on information in the media?	20 (4.4%)	100 (21.7%)	280 (60.9%)	60 (13.0%)	2.82 ± 0.70	
Health promotion	(13) —find out what activities are good for your mental well-being?	0 (0%)	96 (20.9%)	252 (54.8%)	112 (24.3%)	3.03 ± 0.67	3.08 ± 0.67
	(14) —understand advice on health from family members or friends?	4 (0.9%)	40 (8.7%)	288 (62.6%)	128 (27.8%)	3.17 ± 0.60	
	(15) —understand information in the media on how to get healthier?	8 (1.7%)	84 (18.3%)	268 (58.3%)	100 (21.7%)	3.00 ± 0.68	
	(16) —judge which everyday behavior is related to your health?	4 (0.9%)	80 (17.4%)	236 (51.3%)	140 (30.4%)	3.11 ± 0.70	
Total							3.04 ± 0.70

^*^Mean and standard deviation are shown with the degree of perceived difficulty in each situation proposed in the questionnaire (very difficult = 1, difficult = 2, easy = 3 and very easy = 4).

Logistic regression analysis to predict the relative association of specific variables with HL showed that older age (OR 1.11 CI95 1.06–1.16) was associated with a sufficient level of HL. Female nursing students were 69% less likely to have sufficient HL than male nursing students (OR 0.31 CI95 0.15–0.64) and smokers were 38% less likely to have sufficient HL than male non-smokers (OR 0.62 CI95 0.38–0.99). Participants who lived alone were 0.14 times less likely to have sufficient HL than those who lived with their family (OR 0.14 CI95 0.05–0.37). Finally, participants with a moderate screen-time (≤4 h per day) were 2.5 to 3 times more likely to have sufficient HL than those who spent 5–7 h per day and ≥ 8 h per day. The predictive capacity of the multivariate logistic regression model for the probability of a sufficient level of HL was 23.5% (*R*^2^ = 0.235) (Table 3).

**Table 3:** Multivariate logistic regression analysis^*^ to predict the probability of sufficient level of HL.

	B (standard error)	Adjusted OR	CI 95%	*p*-value
Age (years)	0.10 (0.02)	1.11	(1.06–1.16)	0.000
Gender				
Male (reference)		1	–	–
Female	−1.18 (0.37)	0.31	(0.15–0.64)	0.001
Living arrangement				
Living with family (reference)		1	–	–
Living alone	−1.98 (0.50)	0.14	(0.05–0.37)	0.000
Living with mates	0.12 (0.25)	1.13	(0.69–1.84)	0.630
Employment status				
Unemployed (reference)		1	–	–
Employed	−0.18 (0.41)	0.83	(0.37–1.86)	0.659
Perceived general health				
Bad health (reference)		1	–	–
Good health	0.06 (0.48)	1.06	(0.42–2.70)	0.904
Chronic disorder				
No (reference)		1	–	–
Yes	−0.06 (0.29)	0.94	(0.53–1.65)	0.823
Health care utilization (last year)				
0–1 visits (reference)		1	–	–
2–7 visits	−0.38 (0.31)	0.69	(0.38–1.25)	0.218
≥8 visits	0.62 (0.42)	1.85	(0.82–4.19)	0.138
Self-medication last-week				
No (reference)		1	–	–
Yes	−0.20 (0.29)	0.82	(0.46–1.45)	0.495
Tobacco use				
No smoker (reference)		1	–	–
Smoker	−0.48 (0.24)	0.62	(0.38–0.99)	0.046
Alcohol consumption				
Never or occasionally (reference)		1	–	–
At least once a week	0.21 (0.26)	1.23	(0.74–2.04)	0.418
Screen-time				
≤4 h per day (reference)		1	–	–
5–7 h per day	−1.13 (0.32)	0.32	(0.17–0.61)	0.000
≥8 h per day	−0.89 (0.37)	0.41	(0.20–0.85)	0.016
Diet quality				
Healthy (reference)		1	–	–
Inadequate	−0.99 (0.61)	0.37	(0.11–1.23)	0.106
Need changes	−0.34 (0.50)	0.71	(0.27–1.89)	0.494
Physical activity				
Medium (reference)		1	–	–
Low	0.31 (0.26)	1.37	(0.81–2.30)	0.236
High	−0.33 (0.31)	0.72	(0.40–1.31)	0.285

^*^Nagelkerke’s *R*^2^ = 0.235; Hosmer-Lemeshow test: *p* = 0.000.

## Discussion

4.6 out of 10 nursing students showed inadequate or problematic HL. These figures are slightly higher than those reported in previous studies on nursing students (19, 20). In any case, those obtained by the general Spanish population in the European Health Literacy Survey (14) in which 58.5% of subjects showed inadequate or problematic HL. Small difference if we take into account that nursing students are the future health trainers of the population. These findings suggest an increase in HL content in nursing programs. In this sense, previous studies suggest that students assigned to training programs in which HL has a greater weight have greater training (17, 28, 29). Similarly, the integration of specific training pills in HL in the curriculum of future nurses seems to be effective (30–32).

In our sample, the predictors of insufficient HL were female gender, age, living alone, and certain unhealthy lifestyles. Most previous studies carried out on nursing students have not found significant differences in HL between the sexes (20, 29). Our hypothesis is that the asymmetry of our sample (only 13.9% of men) is probably conditioning the significant influence of gender on HL. This hypothesis is supported by the fact that there were no significant differences in the mean values of the HLS-EU-Q16 scores for men and women, 13.3 ± 2.1 and 12.8 ± 2.6, respectively.

The available literature suggests the existence of a direct linear relationship between the time spent in nursing studies and the level of HL (17, 33, 34). As a general rule, age is an indirect indicator of the academic year. Expanding on this idea, in our study older age was a predictor of sufficient HL.

In our sample, living alone was one of the factors associated with lower HL. This is a relationship that has not been analyzed in the university population but has been previously observed both in the general population (35, 36) and in specific patient populations (37, 38). The main reason for this may be the lack of social support for health that can be perceived by people who live alone (35).

Previous studies have previously linked HL with Health-Promoting Behaviors (7, 39, 40). For example, a low HL would be associated with smoking and its relapses (41, 42). In our study, being a smoker was inversely associated with the probability of having sufficient HL. In nursing students, it is unlikely that the cause of smoking is ignorance of the effects and complications associated with tobacco use. In fact, in our sample, the HL dimension with the highest score was that of “Health promotion” competencies. Thus, our hypothesis is that, beyond mere knowledge of the consequences of smoking, HL has a mediating effect on other parameters of the Health Belief Model. Along these lines, Panahi et al. (43) observed significant correlations in the university population between HL and the perceived susceptibility and perceived severity of smoking; and self-efficacy for the Adoption of Smoking Preventive Behavior.

The relationship between HL and physical activity has been previously tested. In a systematic review of 2020 (44) it was observed that most previous observational studies found a positive and significant association between high HL and high levels of physical activity. However, this association has not been demonstrated in intervention studies, in which an HL-promoting intervention has not led to an increase in physical activity (45). In our sample, HL was not related to greater physical activity but was inversely related to sedentary lifestyle, evidenced by screen-time. This association (HL and screen-time) has not been specifically studied before in university populations. Although it has been reported in previous studies on the adolescent population (46, 47). Our findings do not suggest a significant association between diet quality and HL in nursing students. This is in contrast to previous results observed in specific pregnant (48) or clinical (49) populations. However, this is not necessarily the case in the general population (50). We hypothesize that, in the absence of a specific motivation such as an illness, an adequate level of HL is not a determining factor for the adoption of changes in certain health-related behaviors with long-term effects such as sedentary lifestyle and unhealthy diet. In the case of nursing students, who are assumed to have adequate knowledge about sedentary lifestyle and diet, the perception of risk in the long term may not be a sufficient motivation for physical activity and the adoption of a healthy diet.

To our knowledge, this is the first study that analyzes the level of HL and its associated factors in a large sample of Spanish nursing students. Several factors lead us to believe that our results may be representative of the population of Spanish nursing students. The significant size of our sample, with a gender and age profile consistent with that existing in Spanish training programs, the use of standardized data collection procedures, the use of the HLS-EU-Q16, a validated and extended tool that allows easy comparison with other populations, and the plausibility of the established associations support this presumption. However, this research has some limitations that should be pointed out. Its cross-sectional design precludes any causal conclusion. Longitudinal studies may provide a better basis for understanding the associations observed in this study, especially regarding how HL may mediate between social determinants, lifestyles, and health. In addition, and despite the fact that the HLS-EU-Q16 is a widely used and recommended tool, this research does not include any objective element in the evaluation of functional HL, and is therefore subject to reporting bias (51). In this study, the academic year, a factor that could potentially be associated with HL in nursing students, was not included in the analysis. This was a voluntary decision by the research team based on two fundamental issues. Firstly, in our environment a considerable number of students repeat subjects or are partially enrolled in nursing studies, so it is common to find students with 3 or 4 years of permanence in the University taking subjects from the 1st academic year. Secondly, Spanish educational legislation allows the validation of subjects based on previous studies, not necessarily from Higher Education, by the students. Thus, it is common to find students in their 1st year of access to Nursing studies, taking subjects from the 3rd or 4th academic year. For these reasons, the academic year variable could be, in our setting, more of a confounding factor than an adequate predictor of HL.

## Conclusion

The findings of this study suggest that the HL of nursing students is low. Factors associated with problematic HL were younger age, living alone, and certain unhealthy lifestyles (smoking and prolonged screen-time).

Two main reasons suggest the need for an effort to promote HL among nursing students: 1. It is a young-adult population, a crucial time in the acquisition and reinforcement of health-related habits. Thus, in the university environment and period, previously learned patterns can be cemented, or new patterns can be learned that affect health in the short and long term (52) and 2. nurses are the health professionals with the greatest specific weight in education for health and the approach to HL problems aimed at promoting health. In this way, the greater the acquisition of HL during undergraduate education, the more competent their subsequent performance will be. This fact will result in more empowered patients and communities (53).

Under this prism, nursing educators should promote HL awareness in students and emphasize the importance of patient empowerment through HL in the curriculum.

## Data availability statement

The original contributions presented in the study are included in the article, further inquiries can be directed to the corresponding author/s.

## Ethics statement

The studies involving human participants were reviewed and approved by the Clinical Research Ethics Committee of Aragón. The patients/participants provided their written informed consent to participate in this study.

## Author contributions

ER-A: conceptualization, analysis, and investigation. ER-A and JG-L: methodology. IA-S: software. AC-R and AR-C: resources and data curation. IA-S and ER-A: writing—draft. ER-A, JG-L, and EE-S: writing—reviewing and editing. AR-C: supervision. RJ-V: funding acquisition. All authors contributed to the article and approved the submitted version.

## Conflict of interest

The authors declare that the research was conducted in the absence of any commercial or financial relationships that could be construed as a potential conflict of interest.

## Publisher’s note

All claims expressed in this article are solely those of the authors and do not necessarily represent those of their affiliated organizations, or those of the publisher, the editors and the reviewers. Any product that may be evaluated in this article, or claim that may be made by its manufacturer, is not guaranteed or endorsed by the publisher.
